# Photophysics and Electrochemistry of Biomimetic Pyranoflavyliums: What Can Bioinspiration from Red Wines Offer?

**DOI:** 10.3390/photochem2010003

**Published:** 2022-01-06

**Authors:** Eli Misael Espinoza, John Anthony Clark, Mimi Karen Billones, Gustavo Thalmer de Medeiros Silva, Cassio Pacheco da Silva, Frank Herbert Quina, Valentine Ivanov Vullev

**Affiliations:** 1 Department of Chemistry, University of California, Riverside, CA 92521, USA; 2 Department of Bioengineering, University of California, Riverside, CA 92521, USA; 3 Department of Biology, University of California, Riverside, CA 92521, USA; 4 Instituto de Química, Universidade de São Paulo, Avenida Lineu Prestes 748, Cidade Universitaŕia, São Paulo 05508-900, Brazil; 5 Department of Biochemistry, University of California, Riverside, CA 92521, USA; 6 Materials Science and Engineering Program, University of California, Riverside, CA 92521, USA

**Keywords:** pyranoflavyliums, anthocyanins, pyranoanthocyanins, dye-sensitized solar cells

## Abstract

Natural dyes and pigments offer incomparable diversity of structures and functionalities, making them an excellent source of inspiration for the design and development of synthetic chromophores with a myriad of emerging properties. Formed during maturation of red wines, pyranoanthocyanins are electron-deficient cationic pyranoflavylium dyes with broad absorption in the visible spectral region and pronounced chemical and photostability. Herein, we survey the optical and electrochemical properties of synthetic pyranoflavylium dyes functionalized with different electron-donating and electron-withdrawing groups, which vary their reduction potentials over a range of about 400 mV. Despite their highly electron-deficient cores, the exploration of pyranoflavyliums as photosensitizers has been limited to the “classical” *n*-type dye-sensitized solar cells (DSSCs) where they act as electron donors. In light of their electrochemical and spectroscopic properties, however, these biomimetic synthetic dyes should prove to be immensely beneficial as chromophores in *p*-type DSSCs, where their ability to act as photooxidants, along with their pronounced photostability, can benefit key advances in solar-energy science and engineering.

## 1. Introduction

Due to their inherent photostability and a broad variety of optical and electronic properties, naturally occurring plant dyes are an important source of inspiration for structure–function relationships that drive the development of a plethora of applications in photonics, optoelectronics, energy science, and biomedical engineering [1–5]. For example, since Hans Fischer elucidated the structural motifs of chlorophylls and synthesized haemin, for which he received the 1930 Nobel Prize in chemistry, synthetic porphyrins have been responsible for crucial advances in science and engineering. Porphyrin structures have served as the basis for dye-sensitized solar cells (DSSCs) with the highest efficiencies [6,7] and other bioinspired charge-transfer (CT) and energy-conversion systems [8–13]. Porphyrins are some of the most indispensable photoredox catalysts [14–16] and photodynamic-therapy agents [17,18].

**Chart 1.**
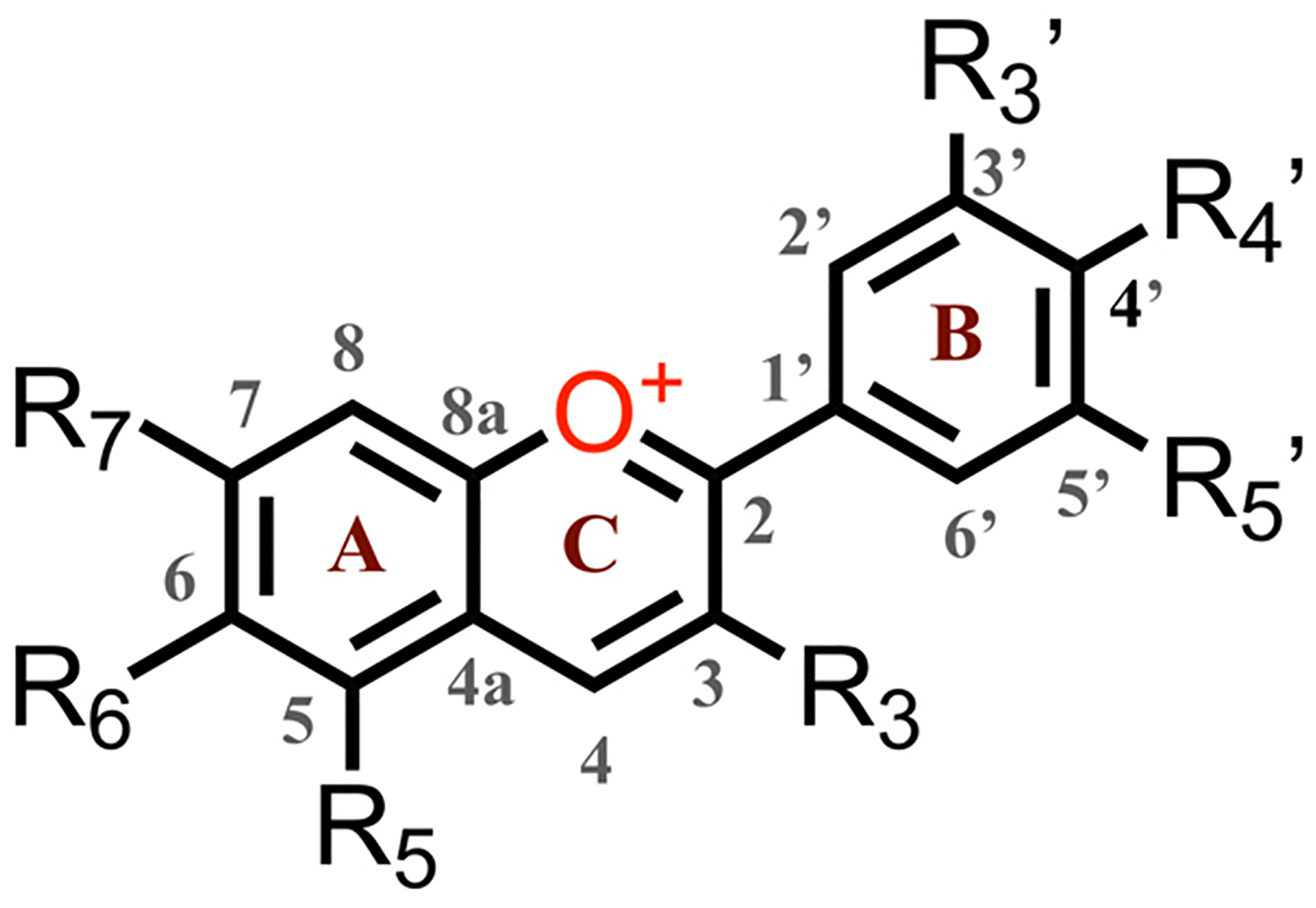
Natural anthocyanins. The seven substituents vary between −H, −OH, −OCH_3_, and −O-carbohydrate.

Among natural dyes and pigments, anthocyanins comprise a large class of polyphenols that produce a diverse range of colors in a variety of plants, fruits and vegetables (Chart 1) [2,5,19]. As flavonoids, anthocyanins also possess antioxidant capabilities based on their redox properties that, along with their photostability and broad absorption in the visible spectral region, make them promising light-sensitizers for organic photovoltaic (PV) solar cells [20–23].

Natural anthocyanin dyes are synthetically challenging and isolation of single dyes proves to be equally challenging [21,22]. Therefore, many anthocyanin photosensitizers in DSSCs are a cocktail of dyes absorbing in different wavelength ranges [22,24]. Such mixtures of chromophores, however, which also contain colorless compounds that passivate the surface of TiO_2_, result in PV cells with power conversion efficiencies (*η*) of only about ~0.6% [21]. Despite the rather miniscule *η* of the anthocyanin DSSCs, the reported half-lifetime of almost 2000 days [24] attests to the amazing photostability of these natural dyes.

**Scheme 1.**
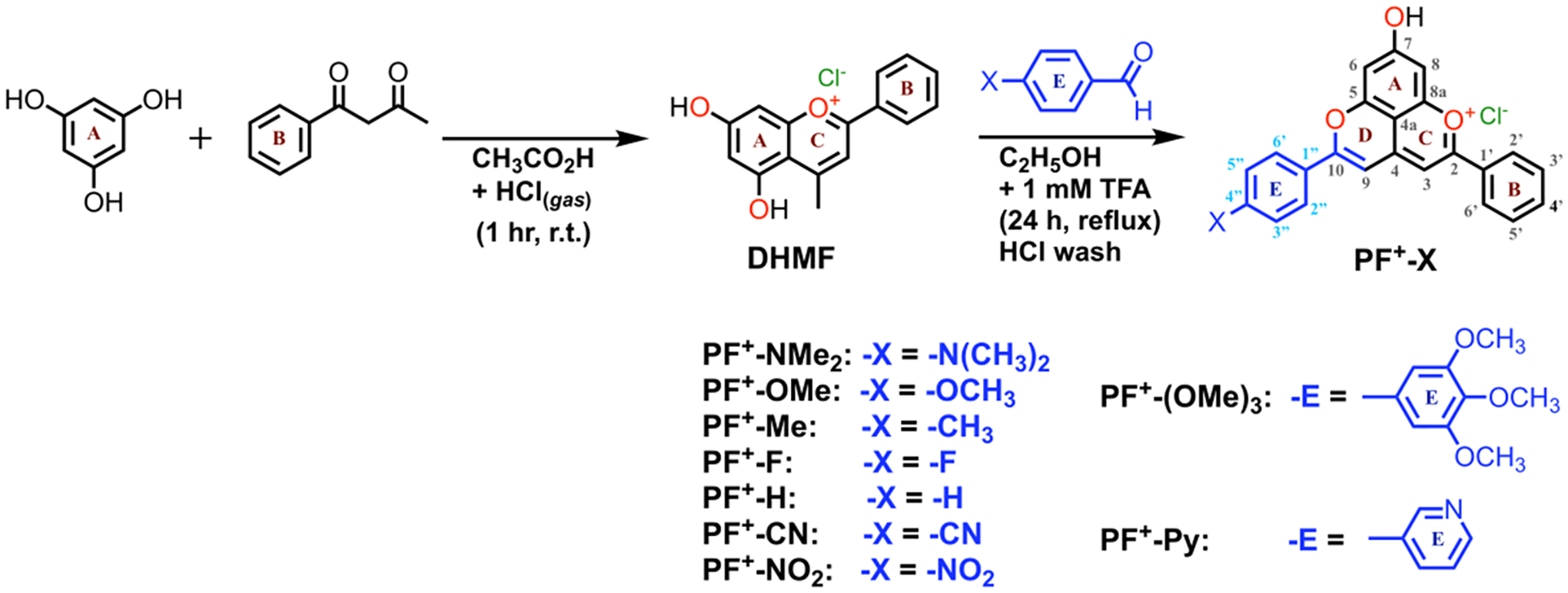
Synthesis of pyranoflavylium dyes, **PF**^**+**^**-X**.

The anthocyanins are most stable under acidic conditions, and lose color at pH greater than 3 due to the nucleophilic attack of water to form hemiketals and chalcones [25,26]. One way to suppress this pH color dependency of the anthocyanins is to convert them chemically to pyranoanthocyanins, containing the pyranoflavylium cation (**PF**^**+**^) chromophore, a reaction that occurs with the natural dyes during the maturation of red wines. The formation of the pyran ring D (Scheme 1) suppresses their susceptibility to elevated pH. The addition of ring E provides a means to modulate the electronic properties of these dyes and produce synthetic **PF**^**+**^ conjugates with donor–acceptor and donor–π-bridge–acceptor architectures, which are essential for DSSCs with improved *η* [19,25,26].

As revolutionary as plant dyes and pigments may prove to be for many applications, basing large-scale technologies on natural products, obtained mostly from living systems, can have devastating impacts on the environment that offset the benefits from a viable solar-energy industry. Recent history shows numerous examples of how cultivating, planting and harvesting large-scale crops for obtaining desired natural products can irreversibly damage ecosystems and adversely interfere with the balance in the biosphere. Synthesis of analogs of such natural dyes offers an agreeable alternative where, for example, fossil materials are converted to useful products, rather than burned as fuels. Furthermore, organic synthesis provides access to biomimetic and bioinspired structures allowing the pursuit of properties and functionalities that the natural analogues do not offer [27–32].

Due to an additional *O*-glycosyl substituent at position 3, natural pyranoflavylium dyes prove to be quite complex to purify and/or modify, rendering them impractical as precursors for pursuing viable PV applications. This challenge has motivated the search for alternative routes to **PF**^**+**^ chromophore structures. Because the sugar groups at position 3 do not affect the photophysical properties of **PF**^**+**^ chromophores or their anthocyanin precursors, the five-aromatic-ring **PF**^**+**^ structures can be more expeditiously synthesized with no substituent at position 3. Strategies employing anthocyanin precursors with a methyl group at position 4, i.e., 5,7-dihydroxy-4-methylflavylium cations (DHMFs, Scheme 1, vs. natural anthocyanins, Chart 1), permit relatively facile preparation of a myriad of **PF**^**+**^ analogues in large quantities and in good yields (Scheme 1) [25,26].

The initial examples of **PF**^**+**^ dyes serving as photosensitizers in liquid-junction PV devices resorted to carboxyl derivatives of these chromophores, obtained via chemical modification of natural anthocyanins extracted from the skins of red grapes and from blackberry fruits [33]. While the reported fill factor (FF) values of the resultant *n*-type dye-sensitized solar cells (*n*-DSSCs) ranged between 0.38 and 0.49, their power-conversion efficiencies, *η*, were more than three-orders-of-magnitude smaller than the *η*-values of DCCSs using the ruthenium dye N719 as the sensitizer [33]. Further improvements of **PF**^**+**^**-based** *n*-DSSCs elevated their *η* to about 0.2% to 1.6% with FF values around 0.6 [34,35]. These studies also revealed that making the peripheral B or E rings electron-rich improved *η*. This motivated the pursuit of DSSCs employing synthetic **PF**^**+**^ derivatives with a strongly electron-donating group attached to the E ring (Scheme 1). An amine substituent on ring E introduces a pronounced CT character into the excited states and causes a more than 100-nm bathochromic shift in the **PF**^**+**^ absorption, extending it into the red spectral region. Employing such dimethylamino-substituted **PF**^**+**^ dyes in DSSCs increased the efficiencies, *η*, to about 2.5% with FF around 0.45 to 0.6 [19]. In designing the **PF**^**+**^ photosensitizers, it was expected that carbohydrate substituents, as well as carboxyl and hydroxyl groups on the B and E rings would be essential for binding the dyes to the semiconductor oxide surfaces. Nonetheless, these recent studies unequivocally showed that removing the carbohydrates from the **PF**^**+**^ rings, and replacing a catechol B ring with a simple phenyl ring (as in **PF**^**+**^**-NMe**_**2**_), considerably improved *η* of the DSSCs [19]. The key feature that ensures high efficiencies is the tertiary amine on ring E [19]. These findings confirm that synthetic biomimetic **PF**^**+**^ dyes lacking the substituents of their natural counterpart, but containing “non-native” groups, such as a tertiary amine, have the potential to serve as metal-free light-sensitizers for liquid-junction PV devices.

Herein, we survey the photophysical and electrochemical properties of a series of synthetic **PF**^**+**^**-X** dyes with different electron-donating and electron-withdrawing groups, **X**, on ring E (Scheme 1). Although the dihedral freedom of the biaryl link between rings D and E could potentially weaken the electronic coupling, such that the substituents on ring E would exert only small effects on the electronic properties of the dyes, varying the groups on ring E from strongly electron-donating to strongly electron-withdrawing in fact caused almost a 0.4-V shift in the one-electron reduction potential of the dyes. Correlation analysis with Hammett sigma constants and Swain–Lupton parameters revealed the importance of resonance between the substituent groups and the π-orbitals on ring E in governing their effect on the reduction potentials. We discuss these findings in light of the feasibility of employing bioinspired synthetic **PF**^**+**^ dyes as sensitizers for liquid-junction solar cells.

## 2. Materials and Methods

### 2.1. Materials

All reagents and solvents were purchased from Merck, Fluka Sigma-Aldrich, or Fisher Scientific and used as received. Synthesis of the **PF**^**+**^**-X** compounds was carried out as previously reported [26]. Product identity was confirmed using ^1^H and ^13^C NMR spectroscopy (methanol-d_4_ + 1% TFA-d) and high-resolution mass spectrometry (HRMS). Product purity was examined using reverse-phase high-pressure liquid chromatography (HPLC) equipped with a Promosil C-18 column and photodiode-array detector (eluting at 0.8 mL min^−1^ with a water/acetonitrile gradient with 0.05% formic acid added to the solvents, Merck, HPLC grade).

### 2.2. Methods

The spectroscopic and electrochemical studies employed acetonitrile (MeCN) as a solvent because of its relevance to DCCSs.

#### 2.2.1. Steady-State Optical Spectroscopy

Steady-state absorption spectra were recorded in transmission mode using a JASCO V-670 spectrophotometer (Tokyo, Japan); and steady-state fluorescence spectra were measured with a FluoroLog-3 spectrofluorometer (Horiba-Jobin-Yvon, Edison, NJ, USA) equipped with double-grating excitation and emission monochromators. The experiments were conducted at room temperature using 1-cm quartz cuvettes and all samples were purged with argon for 5–10 min. The absorbance at the excitation wavelengths was kept within the range between 0.1 and 0.2 for recording the spectra used for calculating the emission quantum yields. The fluorescence quantum yields, *ϕ*_f_, were determined by comparing the integrated emission intensities of the samples with the integrated fluorescence of a reference sample with a known fluorescence quantum yield, *ϕ*_f_^(0)^:

(1) $$\phi_{f}=\phi_{f}^{\left(0\right)}\frac{\int F(\lambda )d\lambda (1-10^{-A^{\left(0\right)}\left(\lambda_{ex}\right)}}{\int F^{\left(0\right)}(\lambda )d\lambda (1-10^{-A\left(\lambda_{ex}\right)}}{\left(\frac{n}{n^{\left(0\right)}}\right)}^{2}$$

In Equation (1), *F*(*λ*) is the fluorescence intensity at wavelength *λ*; *A*(*λ*_ex_) is the absorbance at the excitation wavelength; *n* is the refractive index of the medium; and the superscript “(0)” indicates the corresponding quantities for the reference with well-established fluorescence quantum yield. An aqueous solution of fluorescein (pH = 10) served as a reference, *ϕ*_f_^(0)^ = 0.93 [36,37].

#### 2.2.2. Electrochemical Measurements

Cyclic voltammetry was conducted using a Reference 600^™^ Potentiostat/Galvanostat/ZRA (Gamry Instruments, Warminster, PA, USA), equipped with a three-electrode cell, as previously described [38]. A glassy carbon electrode and a platinum wire were used for the working and counter electrodes, respectively. A saturated calomel electrode (SCE) (Gamry Instruments) was used for a reference electrode and it was connected with the cell via a salt bridge. The samples were dissolved in anhydrous MeCN with 50 mM (*n*-C_4_H_9_)_4_NPF_6_ added as a supporting electrolyte. The sample concentration was kept around 100 μM. Increasing the dye concertation to 1 mM and above leads to the formation of visible films on the surface of the working electrode. Prior to recording each voltammogram, the sample was extensively purged with argon. The half-wave potentials, *E*^(1/2)^, were determined from the midpoints between the cathodic and anodic peak potentials for reversible or quasi-reversible voltammograms and from the inflection points of the cathodic waves for irreversible reduction. The anodic and cathodic peak potentials, *E*_*a*_ and *E*_*c*_, respectively, were determined from the zero points of the first derivatives of the voltammograms, i.e., the potentials where *∂I*/*∂E* = 0 at *∂E*/*∂t* = constant. The inflection points were determined from the zero point of the second derivatives of the voltammograms, *∂*^2^*I/∂E*^2^ = 0 at *∂E/∂t* = constant [39]. The second derivatives of reversible and quasi-reversible voltammograms showed that the inflection-point potentials are quite close to the mid-points between *E*_*a*_ and *E*_*c*_, ensuring the reliability of the estimates of *E*^(1/2)^ from the inflection points of the irreversible voltammograms [40]. The voltammograms were recorded at a scan rate of 50 mV/s. To correct for potential drifts in the reference electrode, a voltammogram of a solution of a standard was recorded before and after each set of measurements. Ferrocene served as the standard (*E*^(1/2)^ = 0.45 V vs. SCE for MeCN, 100 mM (*n*-C_4_H_9_)_4_NBF_4_) [38].

The relatively high stability of its singly oxidized form, along with its solubility in lipophilic solvents, makes ferrocene an excellent choice for testing the performance of the reference electrodes in organic electrochemistry. Nevertheless, reporting measured potentials versus the ferrocene redox couple as a reference is fundamentally wrong and may lead to misinterpretation of results [41]. The reference should be as invariant as possible to the medium conditions in the electrochemical cells, and the reduction potential of Fc^+^ strongly depends not only on the solvent polarity, but also on the electrolyte concentration [38]. Furthermore, co-adsorption of ferrocene and the analyte, A, on the working electrode can affect $E_{{\text{Fc}}^{+}|\text{Fc}}{}^{\left(1/2\right)}$ and $E_{{\text{A}}^{+}|\text{A}}{}^{\left(1/2\right)}$ [40]. For calibrating the cell and for testing the performance of the reference electrode, therefore, the voltammograms of ferrocene should be recorded by itself, without any analyte present.

The favorable electrochemical behavior of ferrocene has led to the recommendation for using the Fc^+^|Fc pair as a reference [42–47]. Nevertheless, some of the evidence for the invariance of *E*_Fc+|Fc_^(1/2)^ to the solvation medium involve comparisons with the reduction potential of *bis*(benzene)chromium(II) [42,43,48,49] that has a similar size to that of ferrocenium, warranting similar solvation energy. Concurrently, reports show different reduction potentials of ferrocenium for different media [50–53], indicating that *E*_Fc+|Fc_^(1/2)^ is not really invariant to solvation. The observed fluctuations of $E_{{\text{Fc}}^{+}|\text{Fc}}{}^{\left(1/2\right)}$ that can exceed 200 mV when changing solvent polarity, which corresponds to an effective radius of the charge on the ferrocenium of about 0.26 nm [38]. While such fluctuations in the reference might be admissible for some studies, they are unacceptable for most others.

The liquid-junction potentials (*E*_LJ_) in electrochemical cells [41,54], indeed, contribute to the solvent-induced changes in $E_{{\text{Fc}}^{+}|\text{Fc}}{}^{\left(1/2\right)}$. Ensuring that the liquid junctions of the salt bridges are between miscible electrolytes tend to minimize the contributions from *E*_LJ_ to the recorded voltammograms [54]. Measurements with the same setup comprising a salt bridge between an SCE reference electrode and the sample solutions, show that a decrease in the solvent polarity induces a positive shift in $E_{{\text{Fc}}^{+}|\text{Fc}}{}^{\left(1/2\right)}$ [38] and negative shifts in the reduction potentials of quinones, $E_{\text{Q}|\text{Q}}\cdot^{-(1/2)}$ [55], which is consistent with the Born solvation energy, Δ*G*_B_, of the charged species [38,55,56]. The potential difference $\left(E_{\text{Q}|\text{Q}{}^{•}}{}^{-(1/2)}-E_{{\text{Fc}}^{+}|\text{Fc}}{}^{\left(1/2\right)}\right)$ also increases with lowering the solvent polarity [57], since ferrocenium has a larger effective radius than singly reduced benzoquinones [38,55]. *E*_LJ_ depends on the solvents and on the activities of the ions on both sides of the junction. Thus, *E*_LJ_ shifts the measured $E_{{\text{Fc}}^{+}|\text{Fc}}{}^{\left(1/2\right)}$ and $E_{\text{Q}|\text{Q}{}^{•}}{}^{-(1/2)}$ in the same direction, which is contrary to the observed trends. Furthermore, in an agreement with Δ*G*_B_, an increase in the size of the quinones decreases the medium-polarity effects on $E_{\text{Q}|\text{Q}{}^{•}}{}^{-(1/2)}$ [55]. For the employed electrochemical cells, therefore, the effects of the solvation energy dominate over those of the liquid junctions. Such evidence precludes any solvation invariance of $E_{{\text{Fc}}^{+}|\text{Fc}}{}^{\left(1/2\right)}$, warranting caution in assessing the limitations for the use of the Fc^+^|Fc pair as a reference [58].

## 3. Results

### 3.1. Optical Characteristics

**Figure 1.**
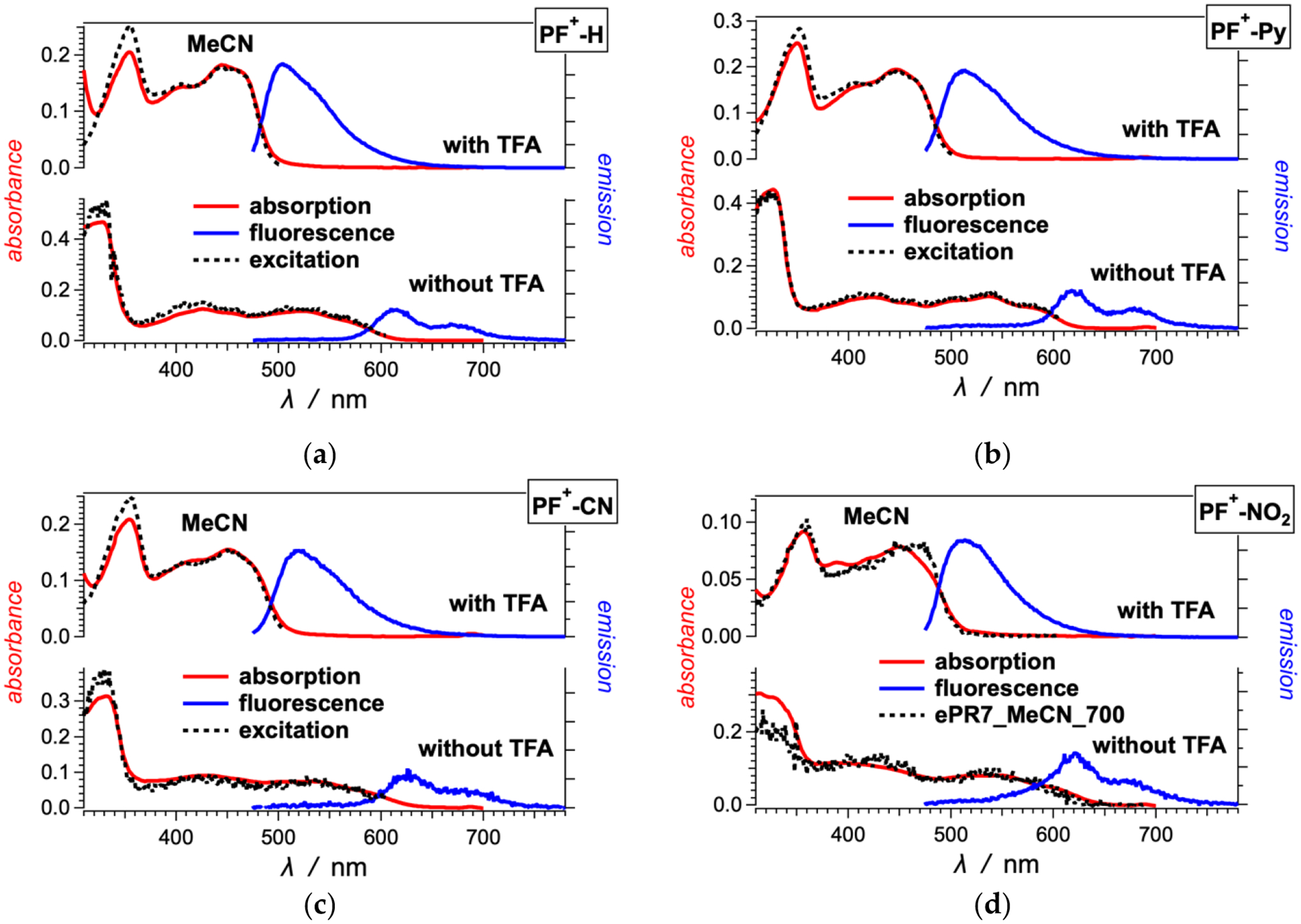
Optical absorption, fluorescence and excitation spectra of (**a**) **PF**^**+**^**-H** and (**b**–**d**) **PF**^**+**^**-X** dyes with electron-deficient ring E (Scheme 1), 10 μM in acetonitrile (MeCN) in the presence and absence of 10 mM trifluoroacetic acid (TFA). (For the emission spectra: *λ*_*ex*_ = 460 nm; for the excitation spectra of **PF**^**+**^**-H**, **PF**^**+**^**-Py** and **PF**^**+**^**-CN**: with TFA, *λ*_*em*_ = 520 nm, and without TFA, *λ*_*em*_ = 615 nm; and for the excitation spectra of **PF**^**+**^**-NO**_**2**_: with TFA, *λ*_*em*_ = 615 nm, and without TFA, *λ*_*em*_ = 700 nm.)

**Figure 2.**
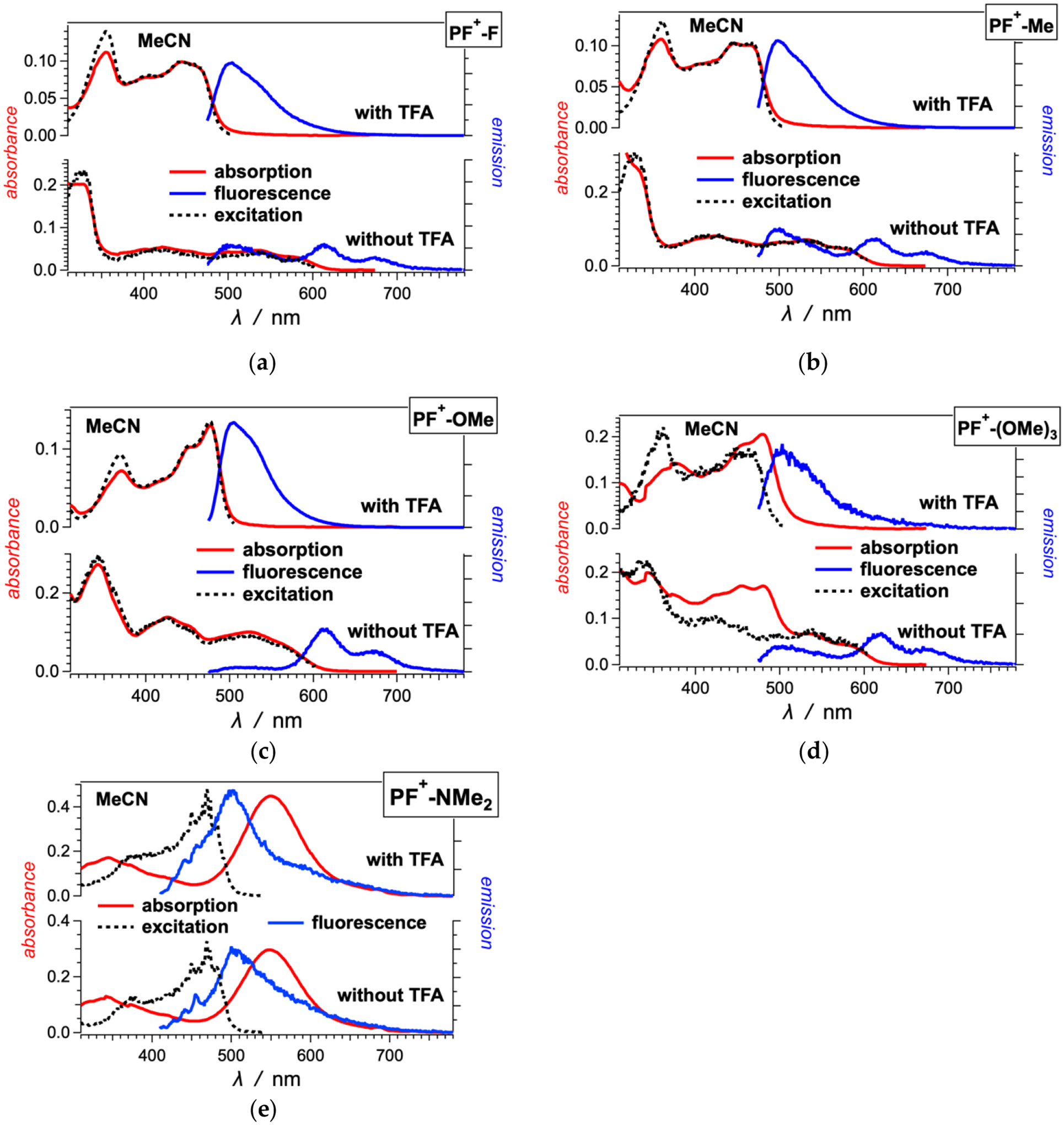
Optical absorption, fluorescence and excitation spectra of (**a**) **PF**^**+**^**-F**, (**b**) **PF**^**+**^**-Me and** (**c**–**e**) other **PF**^**+**^**-X** dyes with electron-donating groups on ring E (Scheme 1), 10 μM in MeCN in the presence and absence of 10 mM TFA. (For the emission spectra: *λ*_*ex*_ = 460 nm; for the excitation spectra of the dyes: with TFA, *λ*_*em*_ = 520 nm, and without TFA, *λ*_*em*_ = 615 nm.)

**Scheme 2.**
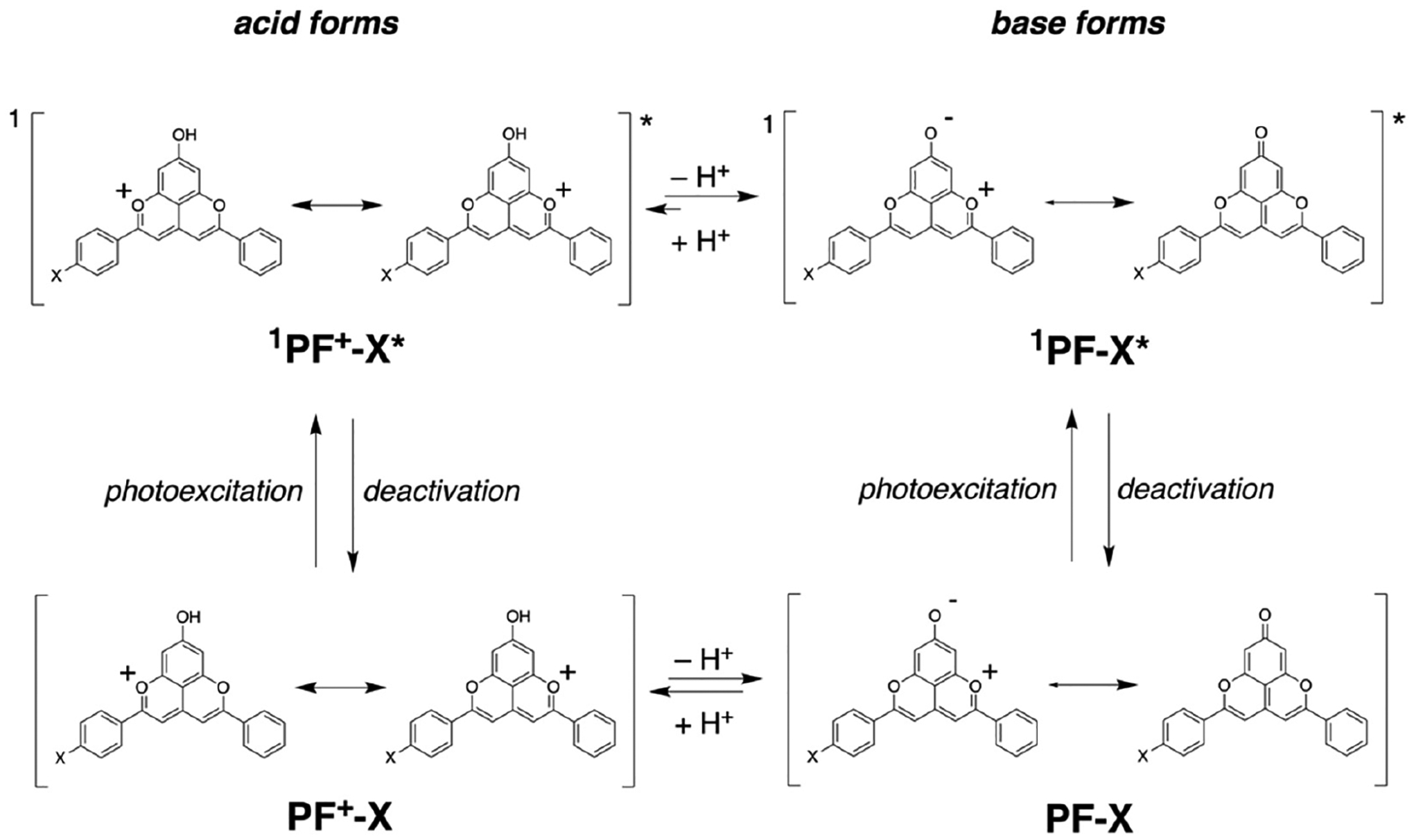
Ground and excited-state prototropic equilibria of the **PF**^**+**^**-X**. dyes.

In the visible spectral region, the **PF**^**+**^**-X** dyes have broad absorption with molar extinction coefficients of around 10,000 to 20,000 M^−1^ cm^−1^, making them a good choice for sensitizers of liquid-junction photovoltaic devices (Figures 1 and 2). The deprotonation of the hydroxyl group on ring A (Scheme 1) has quite a strong effect on the electronic properties of these dyes [59]. Addition of 10 mM trifluoroacetic acid (TFA) ensures the quantitative shift of the equilibrium toward the acid form of the **PF**^**+**^**-X** derivatives in their ground state (Scheme 2). The acid form of most of these dyes exhibits relatively strong greenish fluorescence with emission quantum yields (*ϕ*_*f*_) exceeding 0.01, and zero-to-zero energies $\left(E_{00}\right)$ of about 2.5 eV (Table 1).

The absence of TFA results in a distinct 100-nm bathochromic shift and a decrease in $E_{00}$ to about 2 eV (Figures 1 and 2, Table 1). This shift is consistent with the appearance of the weakly fluorescent base form of the dyes. The absorption spectra in the absence of TFA show contributions from both the acid and base forms of the dyes. The emission spectra of most dyes, on the other hand, show only the long-wavelength fluorescence originating from the base forms of the excited-state dyes (Figures 1 and 2). These observations are considered with the increase in the acidity of the hydroxyl at position 7 upon photoexcitation [59]. While in their ground states, the **PF**^**+**^**-X** dyes exist as mixtures of their acid and base forms, in the excited state, they readily deprotonate and exist predominantly as the excited-state base forms of the dyes.

The spectra of some of the dyes, such as **PF**^**+**^**-(OMe)**_**3**_, **PF**^**+**^**-OMe**, **PF**^**+**^**-Me** and **PF**^**+**^**-F**, also show emission at around 500 nm in the absence of TFA, which originates from their relatively strongly fluorescent acidic forms [59]. Making the E ring electron rich increases the excited state *pK*_a_* of the hydroxyl group on ring A, resulting in only partial deprotonation in the excited state.

The dyes with strongly electron-donating and electron-withdrawing substituents on ring E show a behavior that deviates from the trends of the other derivatives. In MeCN, the amine derivative, **PF**^**+**^**-NMe**_**2**_, shows greenish fluorescence that is shifted hypsochromically from the absorption band at 550 nm (Figure 2e). This 550-nm band is a distinct feature of **PF**^**+**^**-NMe**_**2**_ and has higher molar extinction coefficient than the bathochromic-edge bands of the other **PF**^**+**^**-X** dyes. The excitation spectrum of **PF**^**+**^**-NMe**_**2**_ does not match its absorption at all, and shows that the fluorescence originates from species that absorb at about 470 nm. Addition of TFA does not alter these spectral features of **PF**^**+**^**-NMe**_**2**_ (Figure 2e). Acetonitrile is an immensely weak base and a poor proton acceptor, increasing the *pK*_*a*_ values of acids dissolved in it [61,62]. With the electron-donating amine, therefore, the OH group of **PF**^**+**^**-NMe**_**2**_ remains protonated even in the absence of acid. The electron-rich E ring attached to the electron-poor **PF**^**+**^ rings ensures the formation of a charge-transfer (CT) state, which in this case is optically accessible from the ground state resulting in the 550-nm absorption band.

The CT state does not have noticeable fluorescence and the 470-nm band in the excitation spectrum appears to originate from a small amount of protonated amine of the dye that is responsible for the observed emission. This ammonium derivative, **PF**^**+**^**-N**^**+**^**HMe**_**2**_, absorbs between 400 and 500 nm [63] and has optical features similar to the other dyes with electron-withdrawing groups, such as **PF**^**+**^**-CN** (Figure 1c). The relatively high fluorescence quantum yields of the acidic forms of the **PF**^**+**^**-X** dyes with electron-withdrawing substituents ensure that even traces of **PF**^**+**^**-N**^**+**^**HMe**_**2**_ can yield detectable emission, as observed (Figure 2e).

With an electron-rich E ring, **PF**^**+**^**-(OMe)**_**3**_ also shows CT absorption in acidified MeCN. In the presence of TFA, the excitation and the fluorescence spectra of **PF**^**+**^**-(OMe)**_**3**_ originate from its acidic form (Figure 2d). The bathochromically shifted band of the absorption at 480 nm corresponds to an optical transition from the ground to a CT state (Figure 2d).

### 3.2. Electrochemical Behaviour

#### 3.2.1. Oxidation and Reduction of PF^+^-H

**Figure 3.**
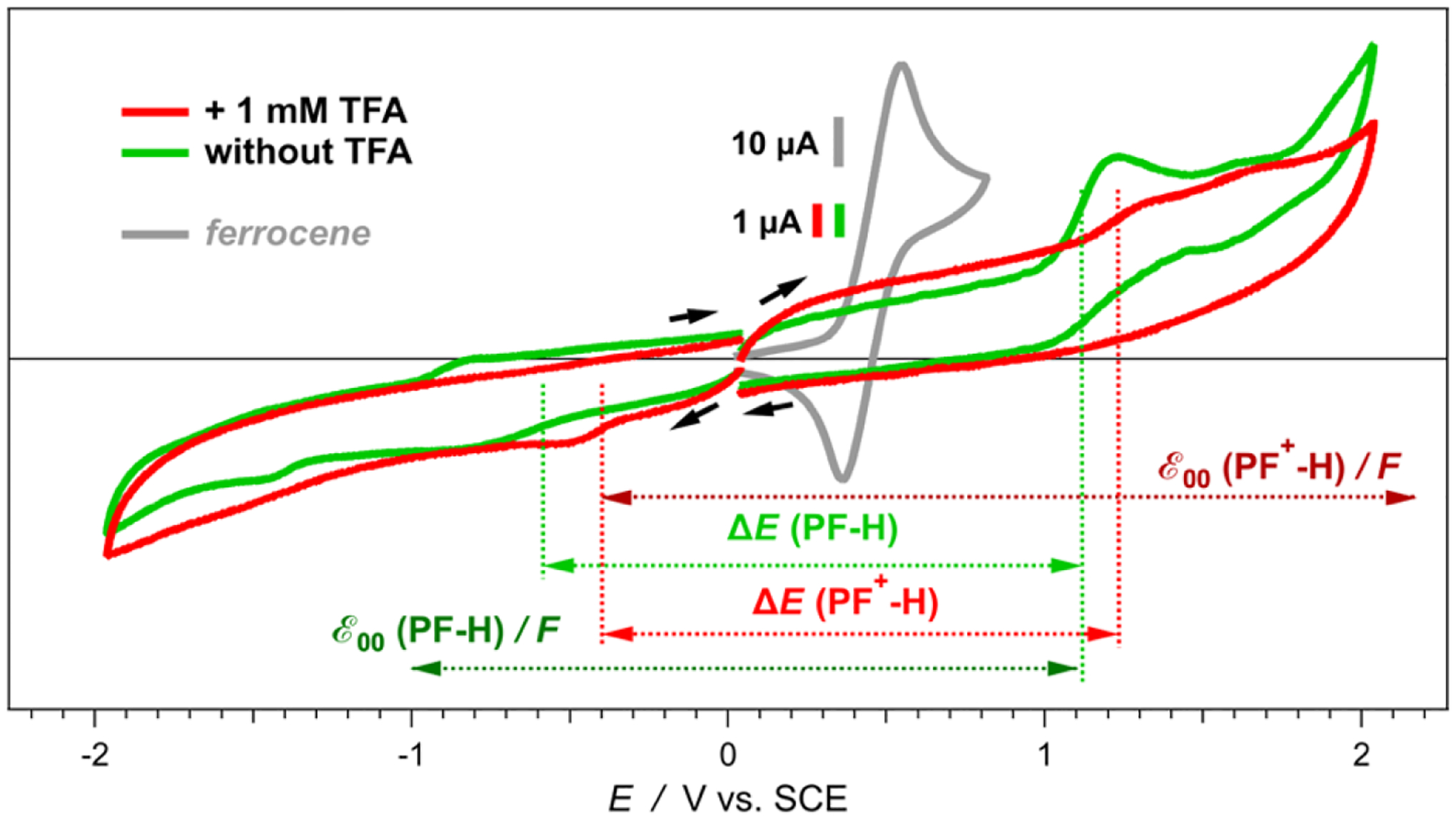
Effects of added trifluoroacetic acid, TFA (1 mM), on the cyclic voltammograms of **PF**^**+**^**-H** in MeCN in the presence of 50 mM (*n*-C_4_H_9_)_4_NPF_6_, *v* = 50 mV s^−1^. The dye concentration was kept at 100 μM. Cyclic voltammograms of ferrocene (1 mM) in MeCN in the presence of 100 mM (*n*-C_4_H_9_)_4_NBF_4_, *v* = 50 mV s^−1^ (under these conditions, $E_{{\text{Fc}}^{+}|\text{Fc}}{}^{\left(1/2\right)}=0.45\text{ V}$ vs. SCE [38]), are recorded before and after each series of electrochemical measurements of the dyes to check for any potential drifts of the SCE reference electrode while immersed in the organic electrolyte solution.

These pyranoflavylium dyes exhibit a complex electrochemical behavior that strongly depends not only on the acidity but also on the ionic strength of the media. For acetonitrile with no TFA added, the cyclic voltammograms of the dye with no substituent on ring E, **PF**^**+**^**-H**, show a well-defined oxidation wave at about 1.1 V vs. SCE (Figure 3). The reduction wave in the absence of TFA, on the other hand, has a small amplitude and spreads between about −0.6 and −1 V vs. SCE (Figure 3). This broad wave is consistent either with reducing multiple species at closely situated potentials, or with multiple one-electron reduction steps of the same species.

**Figure 4.**
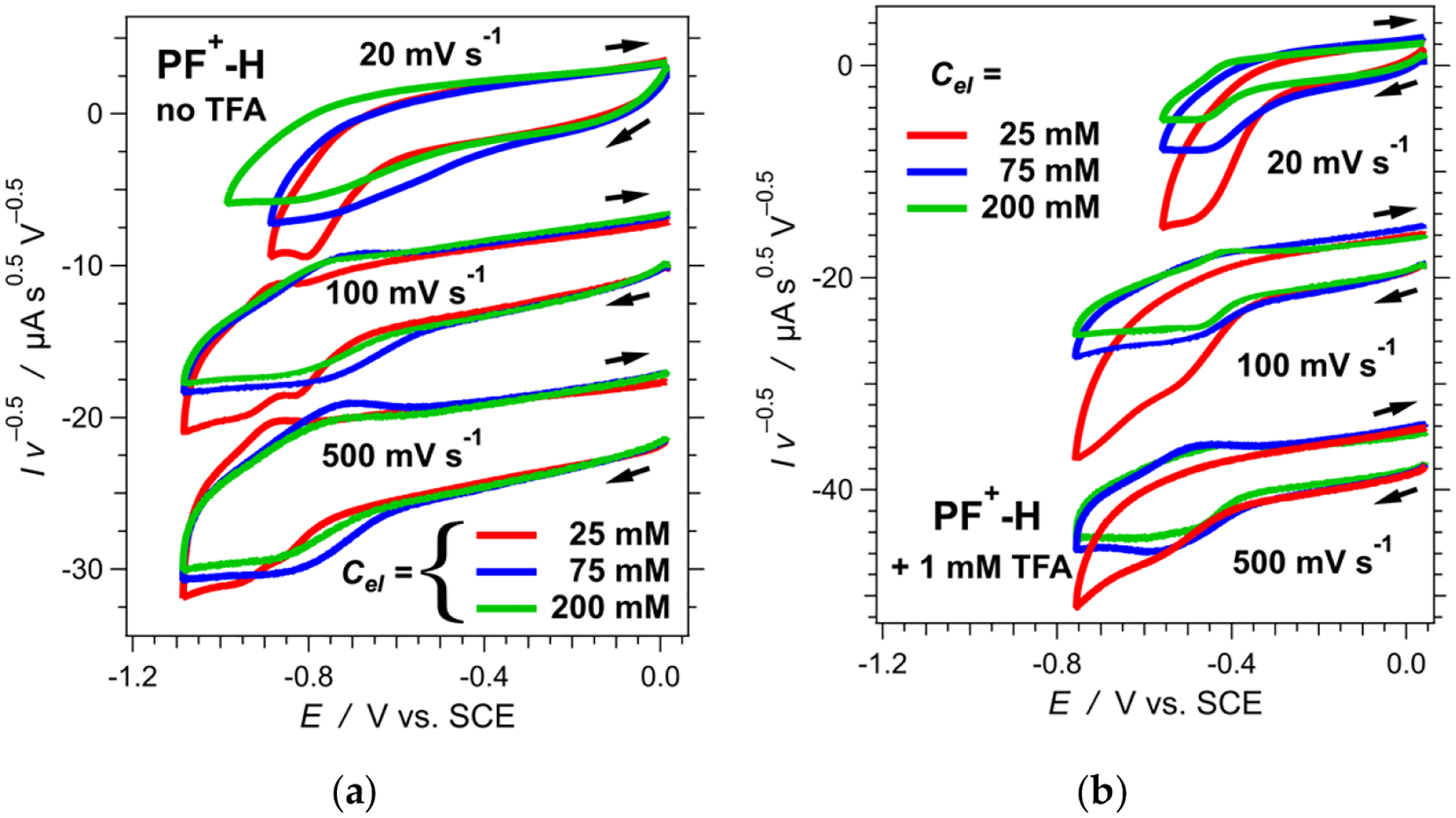
Dependence of the cyclic voltammograms of **PF**^**+**^**-H** on the scan rate, *v*, and the concentration of (*n*-C_4_H_9_)_4_NPF_6_ as the supporting electrolyte, *Cel*, in acetonitrile. Each voltammogram is normalized by the square root of the scan rate. (**a**) Cyclic voltammograms of **PF**^**+**^**-H** with no TFA added. (**b**) Cyclic voltammograms of **PF**^**+**^**-H** in the presence of 1 mM TFA.

An anodic wave in the reduction scans, in the absence of TFA, appears at more negative potentials than the rise of the cathodic signal (Figure 3). Furthermore, the anodic wave is absent when the scan sweeps are reversed closely after the initial growth of the cathodic wave (Figure 4a). These findings suggest that the initial reduction is not reversible. Accumulated products from the consequent reduction steps oxidize after the reversal of the scans at around −1 to −0.9 V vs. SCE.

An increase in scan rates shows some improvement in the reversibility. Changes in the concertation of the supporting electrolyte (*C*_*el*_) also affects the voltammograms quite noticeably (Figure 4a). At *C*_*el*_ = 25 mM, the voltammograms show two distinct cathodic waves, and only the second one manifests some reversibility as apparent from the corresponding anodic peak. At scan rates of 100 mV s^−1^ and higher, a slight increase in *C*_*el*_ “blurs” the two cathodic waves into one broad signal, which has an equally broad anodic counterpart (Figure 4a). A further increase in *C*_*el*_ to about 200 mM broadens the waves and decreases their amplitudes (Figure 4a).

In their ground states, the pyranoflavylium dyes can exist in their positively charged protonated forms, in zwitterionic deprotonated forms and in quinoidal deprotonated forms (Scheme 2). The equilibria between these two charged and one non-charged species are susceptible to the ionic strength of the medium, reflecting the alterations in the appearance of the voltammograms upon changing *C*_*el*_. The rates of proton transfer between these dyes and the solvation media are orders of magnitude faster than the timescales of electrochemical measurements. When the oxidation of one of the species, e.g., with a zero net-charge, occurs at a more negative potential than the oxidation of the positively charged pyranoflavylium, fast reestablishment of the protic equilibrium during the voltage sweep results in a voltammogram with a single anodic peak. That is, oxidizing one of the deprotonated species depletes them from the surface of the electrode. Along with diffusion from the bulk, a quick reach of the protic equilibrium can replenish the depleted deprotonated dye, while decreasing the amounts of the protonated species. These processes preclude the electrochemical detection of species with more positive potentials than the potential of the deprotonated forms that are the easiest to oxidize [64,65]. That is, the fast proton transfer rates during the potential sweeps result in voltammograms that appear as the oxidation of single species, as the observed patterns reveal (Figure 3).

As the optical absorption spectra show, acidifying the medium completely shifts the equilibrium toward the protonated form of the dye (Figure 1a, Scheme 2). Adding TFA: (1) induces positive shifts in the oxidation and the reduction waves; and (2) decreases the amplitude of the oxidation anodic wave, making it similar to the reduction cathodic one (Figure 3). Cyclic voltammograms of TFA solutions with no dyes, recorded under the same conditions, do not show Faradaic signals in the observed region, precluding proton reduction as a possible origin of the observed signals for the acidified dye samples. Furthermore, cyclic voltammograms of freshly prepared **PF**^**+**^**-H** dye sometime show the same reduction cathodic wave at −0.4 V vs. SCE in the absence of TFA. Residual acidity, lingering after the final HCl wash (Scheme 1), can account for such observations.

While increasing the scan rates of the acidified samples leads to some reversibility, it also considerably increases the magnitude of the capacitance current. An increase in the electrolyte concertation, *C*_*el*_, has a stronger impact on improving the reversibility than the increase in the scan rates (Figure 4b). Increasing *C*_*el*_, however, decreases the amplitude of the Faradaic signals.

The dye exists predominantly as a single protonated form in the presence of TFA. Why the amplitudes of the anodic oxidation and the cathodic reduction waves are not as large as those observed for the reduction of the dye in the absence of acid? It is important to consider that the electrochemical oxidation and reduction are interfacial processes. Hence, the Faradaic-current signals strongly depend on the absorptivity of the dye and the position of its molecules in of the double layer on the surface of the working electrode. Differences in the propensities of the electrode material to adsorb the protonated and deprotonated dye renders the comparison between the amplitude in the presence and absence of TFA qualitative at best.

Ascribing the observed initial anodic and cathodic waves in the presence of TFA to the first oxidation and reduction of the protonated dye, **PF**^**+**^**-H**, and in the absence of TFA—to those of the deprotonated one, **PF-H**—suggests huge differences between the electrochemical and optical gaps between the energies of the highest occupied molecular orbital (HOMO) and the lowest unoccupied molecular orbital (LUMO). Koopmans’ theorem correlates measured reduction potentials with the energy levels of the frontier orbitals involved in the interfacial electron transfer (ET) [66,67], and has proven useful for analyzing the photoreduction propensity of pyranoflavylium in DSSCs [35]. Therefore, the difference between the one-electron reduction potentials of a species A, $\Delta E=E_{{\text{A}}^{{•}^{+}}|\text{A}}-E_{\text{A}|{\text{A}}^{{•}^{-}}}$, can serve as an estimate of its electrochemical HOMO-LUMO gap, $E_{\text{E}}=F\;\Delta E$, where the Faraday constant, *F*, relates potentials with energy. Similarly, the zero-to-zero energy, $E_{00}$, represents the optical HOMO-LUMO gap.

Due to Coulombic stabilization between spatially close electron and hole in the excited states, usually, optical HOMO-LUMO gaps are smaller than those obtained from electrochemical potentials or from electron affinity and ionization energy, i.e., $E_{00}≲E_{\text{E}}$.

Nevertheless, certain charged dyes with multiple states of protonation, such as eosin Y and hydroxylated flavyliums, show $E_{00}>E_{\text{E}}$ [68,69]. Similarly, $E_{00}$ of **PF**^**+**^**-H** exceeds its $E_{\text{E}}$ by about 0.9 eV, and of **PF-H**—by about 0.4 eV (Figure 3), which are significant differences.

Employing Koopmans’ theorem for interpretation of electrochemical results warrants a great deal of caution. The experimental measurements yield energies of transitions between states and not energy levels of molecular orbitals [41]. Optical transitions between the ground and excited states with the same multiplicity provide estimates for $E_{00}$. For species with singlet ground states, S_0_, evaluation of $E_{\text{E}}$ relies on transitions between S_0_ and doublet states.

The difference between the optical and electrochemical HOMO-LUMO gaps of **PF**^**+**^**-H** and its deprotonated forms, i.e., $E_{00}>E_{\text{E}}$ (Figure 3), implies that the doublet states of the oxidized and reduced dye under the electrochemical conditions have higher stability than its singlet excited states under the conditions of the optical measurements. Nevertheless, while optical absorption captures the ground-state populations of protonated and deprotonated dyes (Figures 1 and 2), the changes in the p*K*_*a*_ values of the hydroxyls attached to the pyranoflavyliums upon their oxidation and reduction, along with the fast proton transfer, warrant shifts in the potentials of the Faradaic signals during voltammetry measurements. Furthermore, aprotic solvents weaken the strengths of acids (such as TFA and the OH at position 7 (Scheme 1) of the various states of the dye) to a different extent [61,62].

Overall, the protonated 7-hydroxypyranoflavylium dyes are easier to reduce than their deprotonated forms, and the deprotonated 7-hydroxypyranoflavyliums are easier to oxidize than the protonated ones. Therefore, the cathodic waves at the most positive potentials during scans between 0 and −2 V vs. SCE in the presence of TFA can be ascribed to the reduction of the protonated dyes, **PF**^**+**^**-X**. Concurrently, the anodic waves at the most negative potentials during scans between 0 and 2 V vs. SCE in the absence of TFA most plausibly correspond to the reduction of the deprotonated dyes, **PF-X**. The former assignment agrees with reported reduction potentials of flavylium ions that range between about 0 and −0.5 V vs. Ag/AgCl [70] and are on par with reduction potentials of quinones [55,57,71]. The latter is consistent with ascribing the observed anodic waves to the oxidation of quinoidal forms of hydroxyflavylium dyes [35,69]. Therefore, for elucidating the effects of the substituents on ring E of the **PF**^**+**^**-X** dyes (Scheme 1), we focus on the cathodic reduction waves in the presence of TFA and on the anodic oxidation waves in the absence of acid.

#### 3.2.2. Effects of the Substituents on Ring E on the Oxidation and Reduction

**Figure 5.**
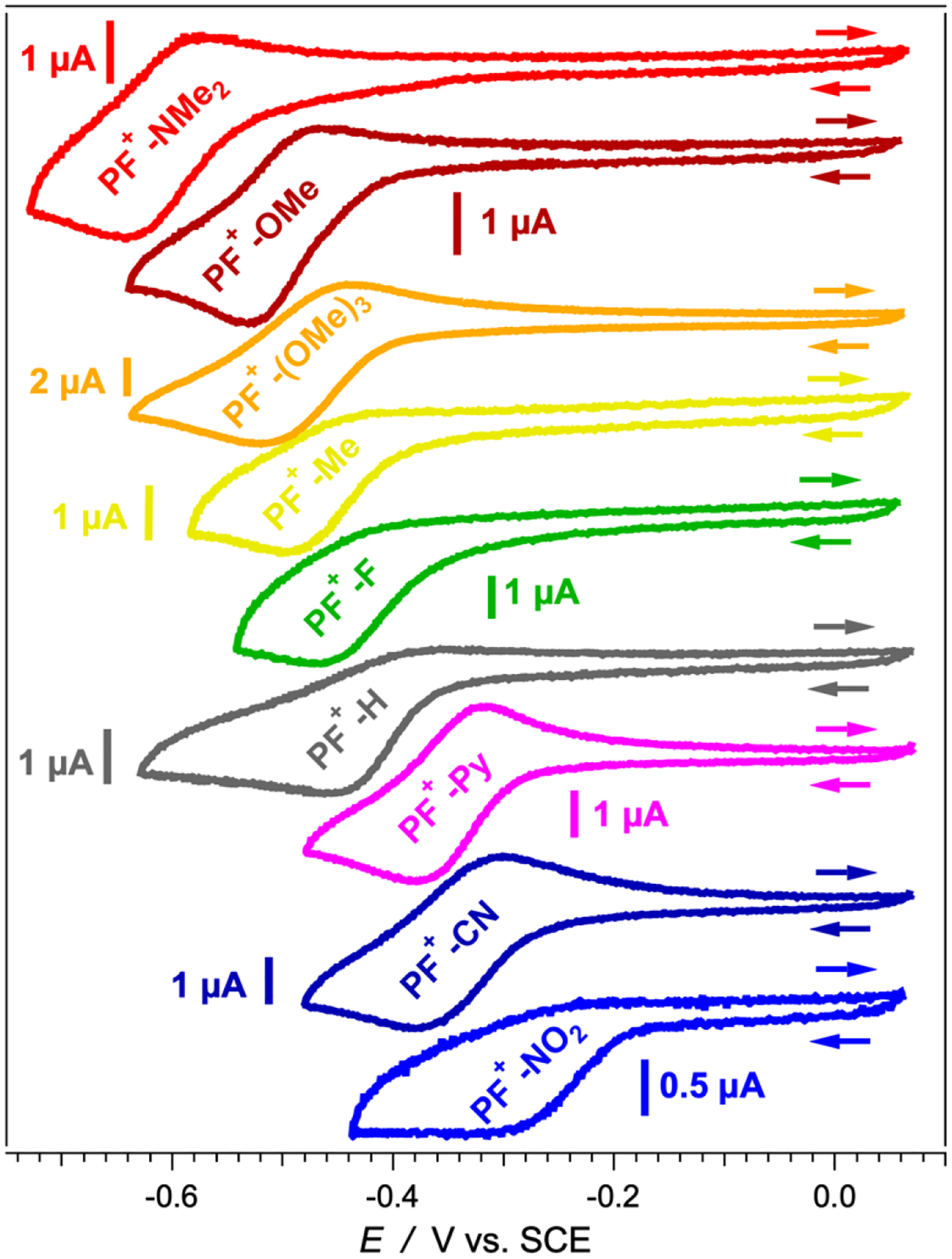
Cyclic voltammograms of **PF**^**+**^**-X** dyes in MeCN in the presence of 50 mM (*n*-C_4_H_9_)_4_NPF_6_, *v* = 50 mV s^−1^, in the presence of 1 mM TFA. The concertation of the dyes was kept at 100 μM.

Voltage scans between 0 and −0.7 V vs. SCE induces one-electron reduction of the acid forms of the dyes, i.e., **PF**^**+**^**-X** + *e*^−^ → **PF·-X**. The cyclic voltammograms of most **PF**^**+**^**-X** derivatives in the presence of TFA reveal partially reversible reduction (Figure 5), indicative of relatively stable radicals, **PF·-X**. The reduction potentials, $E_{{\text{PF}}^{+}|{\text{PF}}^{•}}^{\left(1/2\right)}$, of the **PF**^**+**^**-X** conjugates range between about −0.6 and −0.2 V vs. SCE (Table 2) placing them on par with some of the most electron-deficient organic compounds used as electron acceptors, such as quinones, perylenediimides and nitroaromatics [55,71–78]. The values of their excitation energies, $E_{00}$, make the acidic forms of the **PF**^**+**^**-X** dyes quite potent photooxidants. Most of the photoexcited dyes are thus able to extract electrons from weak electron donors with reduction potentials of their oxidized forms of up to about 2 V vs. SCE (Table 2), which is near the upper limit of the redox window of many solvents of practical importance. Electron acceptors capable of oxidizing species outside the solvent redox window are, of course, to be avoided due to their propensity to induce undesired side reactions involving the solvating medium itself. The photoexcited **PF**^**+**^**-X** dyes are also capable of injecting holes in a wide range of *p*-type semiconductors with valence bands (VBs) above −6 eV vs. the vacuum level (Table 2), such as NiO [79].

**Figure 6.**
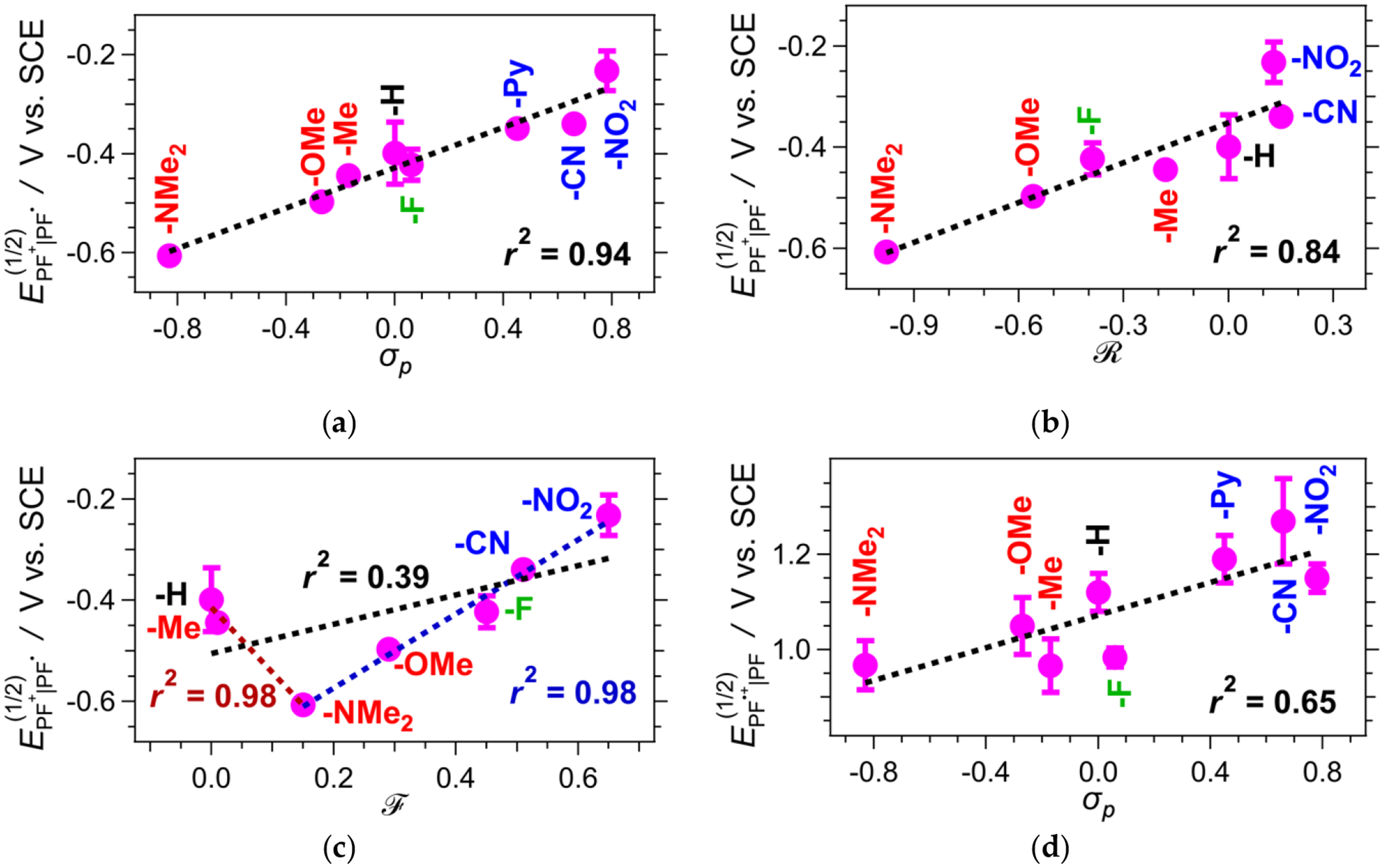
Correlations of the reduction half-wave potentials, *E*^(1/2)^, of protonated **PF**^**+**^**-X** dyes (for MeCN in the presence of 50 mM Scheme 1. mM TFA, Table 2) with: (**a**) the Hammett *para*-substituent constant, *σ*_*p*_, of the substituent, -X, on ring E (for -Py, the reported *σ*_*m*_ of pyridine [86] was employed); (**b**) the Swain–Lupton resonance parameter, R, of -X, accounting for the mesomeric effects via π-conjugation with the aromatic ring; and (**c**) the Swain–Lupton field parameter, F, of -X, accounting for the inductive effect. (**d**) Correlations of the reduction half-wave potentials, *E*^(1/2)^, of the oxidized forms of the deprotonated dyes, **PF**^•**+**^**-X** (with no TFA added, Table 2) with the Hammett *para*-substituent constant, *σ*_*p*_, of the substituent, -X, on ring E. The values of *E*^(1/2)^ were extracted from the average potentials of the anodic and the cathodic peaks of the cyclic voltammograms showing reversible or partially reversible behavior. For the samples exhibiting irreversible reduction, the values of *E*^(1/2)^ were obtained from the inflection point of the cathodic waves [40,84,85]. The dashed lines show the linear correlations, and the corresponding correlation coefficients, *r*^2^, are listed. For (c), in addition to the linear correlation for all samples (in black), the correlation for $F(-X)\leq F\left(-{\text{NMe}}_{2}\right)$ is in red, the correlation for $F(-X)\geq F\left(-{\text{NMe}}_{2}\right)$ is in blue.

The reduction potentials of **PF**^**+**^**-X** exhibit a strong correlation with the corresponding Hammett constants (*σ*_*p*_) of the substituents, **-X**, on ring E (Figure 6a) [80]. As expected, an increase in the electron-withdrawing and a decrease in the electron-donating strength of the substituents enhance the propensity of the dyes to act as electron acceptors. Dissecting this correlation further reveals differences in the contributions from the inductive (along the σ-bonds) and mesomeric (via π-conjugation) effects of the substituents. Likewise, the reduction potentials of **PF**^**+**^**-X** show a strong correlation with the Swain–Lupton resonance primers (R) accounting for the mesomeric effects (Figure 6b) [81]. Conversely, the reduction potentials only show a clear correlation with the Swain–Lupton field parameter (F), which accounts for inductive effects, for the dyes having substituents that can undergo π-conjugation with the aromatic ring, i.e., −NMe_2_, −OMe, −F, −CN and −NO_2_, while **PF**^**+**^**-H** and **PF**^**+**^**-Me** deviate from the correlation (Figure 6c). These correlation analyses suggest that the reduction properties of **PF**^**+**^ are predominantly determined by the mesomeric effects of the substituents on ring E.

The reduction potentials of the oxidized deprotonated dyes, $E_{{\text{PF}}^{+•}|\text{PF}}^{\left(1/2\right)}$, obtained from the voltammograms recorded in the absence of TFA, range between about 1 and 1.3 V vs. SCE (Table 2), and they do not correlate as strongly with *σ*_*p*_ as $E_{{\text{PF}}^{+}|{\text{PF}}^{•}}^{\left(1/2\right)}$ does (Figure 6d). The ground-state deprotonated dyes exist as zwitterionic and quinoidal species (Scheme 2), which have different energies, warranting multiple pathways to their oxidized forms and possible dispersion of the effects of the ring-E substituents on the measured potentials. Conversely, the reduction of the protonated pyranoflavyliums involves only a transition from their cationic to their radical forms. Furthermore, the substituents on ring E could affect differently the spin-density distributions of the singly reduced protonated dyes, i.e., **PF·-X**, and the singly oxidized deprotonated ones, i.e., **PF·**^**+**^**-X**, which can also contribute to the differences in the strength of correlation between *E*^(1/2)^ and *σ*_*p*_ (Figure 6a vs. Figure 6d).

The excitation energies of the deprotonated dyes make them moderately good photoreductants. That is, most of the photoexcited deprotonated dyes can transfer electrons (1) to good acceptors, such as perylenediimides and fullerines [72,73,87,88], with ground-state reduction potentials more positive than about −0.9 V vs. SCE, and (2) to *n*-type semiconductors with conduction bands (CBs) below about −3.8 eV vs. the vacuum level (Table 2). As expected, the dyes with the electron-withdrawing groups, such as **PF**^**+**^**-CN** and **PF**^**+**^**-NO**_**2**_, however, are not as good electron donors as the rest of the pyranoflavyliums.

While these bioinspired positively charged pyranoflavylium dyes are inherently strong electron acceptors, in their deprotonated non-charged quinoidal forms, they are moderately good electron donors. That is, changing the state of protonation of these dyes can switch their role in photovoltaic devices and other charge-transfer systems.

## 4. Discussion

The excited-state potentials of the deprotonated pyranoflavilium dyes place them on par with many well studied photosensitizers for the TiO_2_ anodes of *n*-DSSCs [22,89]. The conduction-band energies of anatase and rutile are about −3.95 eV and −3.7 eV under the vacuum level, respectively [90]. It renders dyes with excited-state reduction potentials of their oxidized forms, i.e., $E_{{\text{A}}^{{•}^{+}}|\text{A}}-F^{-1}\;E_{00}$, more positive than about −0.75 V vs. SCE incapable of injecting electrons into the CB of TiO_2_ [22].

**Figure 7.**
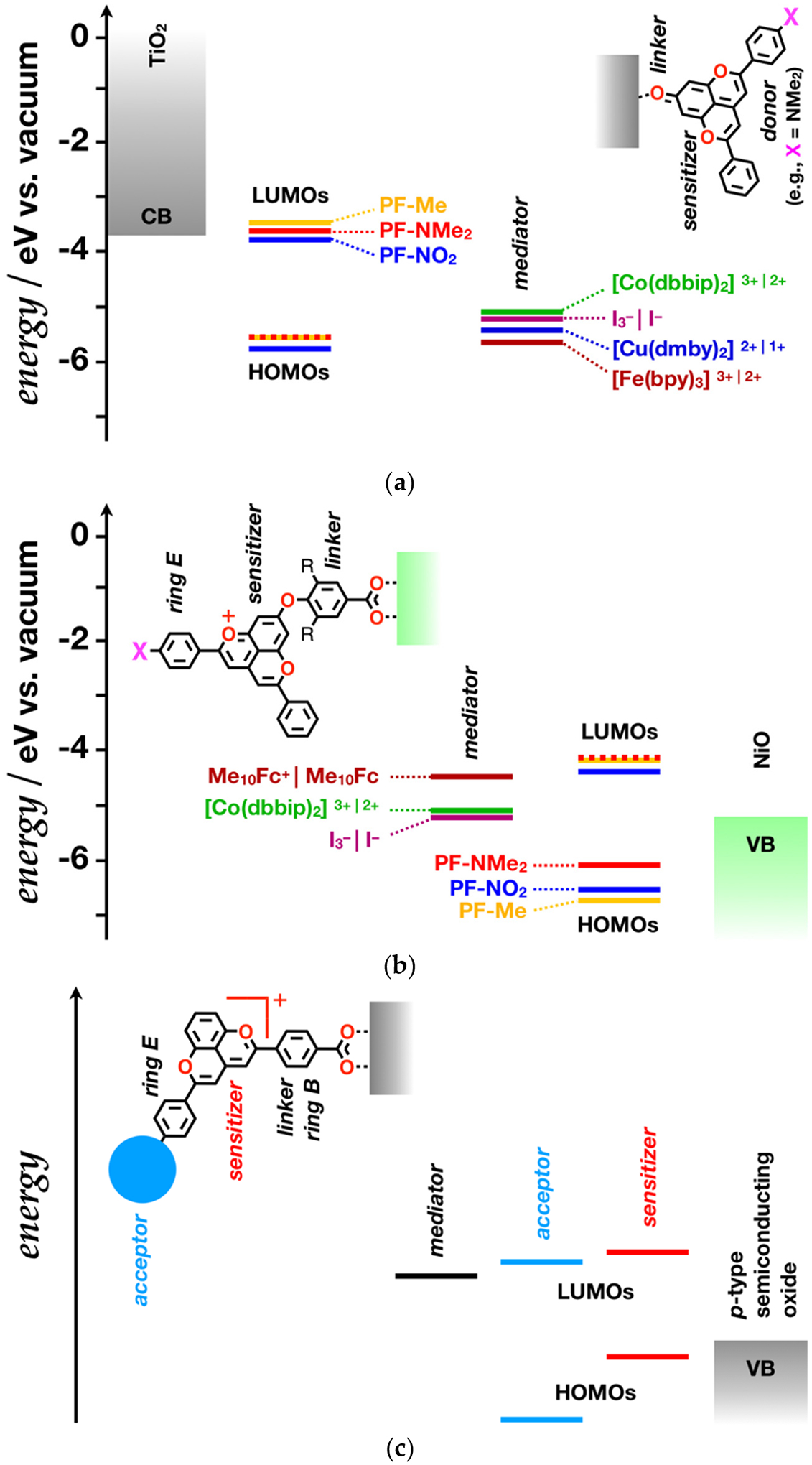
Molecular-orbital (MO) diagrams of DSSCs comprising **PF**^**+**^**-X** derivatives, shown in Scheme 2. (**a**) *n*-DSSC comprising a TiO_2_ photoanode with options for modifying it with different **PF-X** dye sensitizers and using different redox mediators. (**b**) *p*-DSSC employing NiO for a photocathode along with options for different **PF**^**+**^**-X** dyes and redox mediators, assuming minimum perturbation of the MO energy levels upon attaching the aromatic linker to the hydroxyl at position 7. (**c**) A generic diagram of a pyranoflavylium *p*-DSSC with optimized features. (dbbip = di[2,6-bis(1′-butylbenzimidazol-2′-yl)pyridine], dmby = 6,6′-dimethyl-2,2′-bipyridine, bpy = 2,2′-bipyridine, and Me_10_Fc = decamethylferrocene.) For simplicity, the band bending at the interfaces with the dyes is not depicted.

The hydroxyl group on ring A of **PF**^**+**^**-NMe**_**2**_ offers the preferred means for coordinating this dye with the oxide surface, which in turn affects the electronic structure of the **PF**^**+**^ core, comprising rings A, C and D (Figure 7a) [69,91]. Anthocyanin and pyranoflavylium dyes bind to oxide semiconductors via the oxygens of their hydroxyl substituents [69,91]. Depositing these dyes on TiO_2_ surfaces, even under acidic conditions, induces bathochromic shifts that can arise from aggregation or a change in the electronic structure of the **PF**^**+**^ chromophore [19]. The coordination of the **PF**^**+**^**-X** hydroxyls to the titanium ions on the semiconductor surface induces a loss of a proton and the assumption of a quinoidal form [34,35]. Thus, the nature of attachment of these dyes to the TiO_2_ surface ensures that they exist as structures that can photoreduce the semiconductor. In fact, employing **PF**^**+**^**-NMe**_**2**_ as sensitizer produces the highest reported efficiency, *η*, for **PF**^**+**^ *n*-DSSCs [19].

Another important feature of **PF**^**+**^**-NMe**_**2**_, and of other dyes with an electron-rich E ring, is its donor-acceptor architecture, which is key for improving the *η* of *n*-DSSCs. A donor moiety pointing away from the electrode surface pulls the hole farther from the semiconductor, slowing down the geminate charge recombination after the photoinduced electron injection (Figure 7a). Dyes with linker-photosensitizer-donor architectures (where the linker attaches to oxide surfaces) provide some of the highest efficiencies for *n*-DSSCs [6,7,92]. The bioinspired **PF**^**+**^**-X** dyes with electron-donating substituents on ring E, therefore, are promising for sensitizing oxide photoanode when they bind to the electrode surface via their 7-hydoxyl substituent and quinoidal non-charged structures.

The diverse sets of electron-donating substituents on ring E allow for optimizing the driving force for the photoinjection of electrons in the semiconductor. The choice of redox mediators for shuttling electrons from the cathode to the oxidized dye is also key for improving the performance of the DSSCs [93–98]. Selecting the optimal mediators can suppress charge recombination, which enhances the output current. The match between the reduction potentials of the photosensitizers and the mediators should be optimized for improving the voltage output. Triiodide-iodide redox couples offer some of the best thermodynamic and kinetic features for *n*-DSSCs and they have been the primary choice for DSSCs with **PF-X** sensitizers [19,91]. In the last decade, the use of a wide range of metal complexes as mediators have yielded promising results. DSSCs employing Co(III)|Co(II) complexes as mediators exhibit among the largest power-conversion efficiencies for liquid-junction photovoltaic devices [6,7]. Copper (II) chelates tend to be better electron acceptors than I_3_^−^ and can improve the open-circuit voltage of *n*-DSSCs using dyes with low-lying HOMOs [97,99,100], such as the **PF-X** derivatives (Figure 7a). *Tris*-bipyridinium iron(III) chelate is an even better electron acceptor than Cu(II). Using [Fe(bipy)_3_]^3+|2+^ as a mediator pair [101] may prove beneficial for DSSCs with some of the **PF-X** derivatives, but [Fe(bipy)_3_]^2+^ may not be a good enough electron donor to regenerate the photooxidized **PF-X** dye with electron-rich ring E (Figure 7a). These considerations for mediator choices account only for the thermodynamics of the charge-transfer steps. The reorganization energy and the kinetic aspects of the processes, along with the adsorptivity of the involved species, set further challenges and room for improvement of the device performance.

These strategies of developing *n*-DSSCs with **PF**^**+**^ sensitizers do not take advantage of the pronounced capability of these dyes to act as electron acceptors. Reversing the paradigm can truly benefit from the use of such electron-deficient **PF**^**+**^**-X** photosensitizers. As important as *p*-type dye-sensitized solar cells (*p*-DSSCs) are, their development has lagged behind that of *n*-DSSCs [102–104]. In the *p*-DSSCs, photoexcited chromophores inject holes into the cathode. The electrochemical potentials and optical properties of the protonated **PF**^**+**^**-X** dyes render them capable of photoinjecting holes in NiO (with VB at about −5.4 to −5.0 eV under the vacuum level [79]) and a myriad of other *p*-type semiconductors. Therefore, while the performance of *n*-DSSCs with **PF**^**+**^ sensitizers is encouraging, these bioinspired **PF**^**+**^**-X** dyes could prove highly beneficial for the development of *p*-DSSCs.

Anchoring the **PF**^**+**^**-X** derivatives to the oxide *p*-type semiconductors via their 7-hydroxyls, however, transforms them into their quinoidal forms and compromises their propensity to photooxidize [35]. Converting the 7-hydroxyl group to an ether can conserve the positive charge on the **PF**^**+**^ core, while extending the linker for attaching to the oxide surface (Figure 7b). Using an electron-rich π-conjugated moiety as a linker, such as 3,4-alkyl-4hydroxybenzoic acid, can prove beneficial for facilitating the hole transfer to the semiconductor.

To decrease the voltage losses from the regeneration of the reduced dye, the oxidized form of the mediator has to be a worse electron acceptor than I_3_^−^ and Co(III) (Figure 7b). Thermodynamically, the decamethylferrocene couple, i.e., Me_10_Fc^+^|Me_10_Fc [105], presents a promising choice for **PF**^**+**^**-X** *p*-DSSCs (Figure 7b).

As promising as this configuration of **PF**^**+**^**-X** *p*-DSSCs might appear, some of its design aspects require improvements. The VB of NiO lies too high even for the dies with most electron-rich ring E (Figure 7b), which inherently is a source of voltage losses due to unnecessarily large driving forces for the hole photoinjection. A *p*-semiconductor with a VB lying lower than that of NiO may address this issue.

The HOMOs of the **PF**^**+**^**-X** dyes have a node that stretches over the carbon 7 and the hydroxyl on the pyranoflavylium core [106], which inherently weakens the electronic coupling for the hole transfer from the photoexcited dye to the semiconductor. Attaching the dyes to the electrode via the B or E rings, while eliminating the hydroxyl at position 7, offers a means for optimizing pyranoflavylium *p*-DSSCs (Figure 7c). In this case, linker-photosensitizer-acceptor dye architectures will prove essential for moving the electron away from the *p*-oxide surface and suppressing the geminate charge recombination [102]. Placing the surface-anchoring group on one of the rings B and E, leaves the other one to attach an ancillary acceptor to it. It has to be a stronger electron acceptor than the pyranoflavilium core. For example, many benzoquinone derivatives have reduction potentials more positive than −0.4 vs. SCE [71], and can readily relay electrons away from reduced photosensitizers [10,11].

The synthetic scheme of these bioinspired **PF**^**+**^**-X** dyes (Scheme 1) allows preparing derivatives with different substituents on the B and E rings [91], essential for attaining the proposed linker-photosensitizer-acceptor architecture. Eliminating the hydroxyl group on position 7, on the other hand, may affect the electronic structures of these dyes. The nodes of the occupied frontier orbitals of the **PF**^**+**^**-X** derivatives that stretch from carbon 4 to carbon 7 and the oxygen attached to it (Chart 1) [106], however, most likely preclude strong perturbations of the electronic properties of the pyranoflavylium cationic moiety upon removing the hydroxyl from position 7. As an alternative, alkylation of the 7-hydroxyl group, while having minimum effect on the electronic properties of the **PF**^**+**^**-X** dyes, will eliminate its propensity for binding to oxide surfaces.

The ability to vary the electronic properties of these dyes by changing the electron density of ring E offers a range of opportunities to optimize rates of electron and hole injection in the semiconductors of *n*- and *p*-DSSCs at as small diving forces as possible. Employing linker-photosensitizer-donor and linker-photosensitizer-acceptor dye designs suppresses undesired charge recombination. The choice of mediators for shuttling charges between the electrodes is also crucial for optimizing the performance of such devices [93–98]. Therefore, dyes with broadly tunable optical and electrochemical properties, such as **PF**^**+**^**-X** (Scheme 1), are important for advancing the field.

## 5. Conclusions

Inspired by the dye chromophore responsible for the color of red wines, biomimetic **PF**^**+**^**-X** conjugates are immensely attractive for light-harvesting and energy-conversion applications. Their chemical and photochemical stability are impressive [24]. Shifting the paradigm from *n*-type to *p*-type liquid-junction devices, however, is essential for fully exploiting the unique characteristics that pyranoflavylium dyes offer for solar-energy science and engineering.

## Figures and Tables

**Table 1.** Photophysical properties of the **PF**^**+**^**-X** derivatives (Chart 1) for acetonitrile as a solvation medium in the presence and absence of trifluoroacetic acid (TFA).

	Solvent Acidity	*ϕ*_*f*_ × 10^3^^a^	λ_*abs*_/nm ^b^	λ_*exc*_/nm ^c^	λ_*fl*_/nm ^d^	$E_{00}/\text{eV}$ ^e^
**PF** ^**+**^ **-NMe**_**2**_	10 mM TFA	0.96	550	468	500	– ^f^ (1.95)
	No TFA	1.5	549	470	507	– ^f^ (1.94)
**PF** ^**+**^ **-OMe**	10 mM TFA	355	371, 449, 476	369, 449, 476	507	2.54 (2.45)
	No TFA	5.5	342, 426, 520	342, 423, 519	515, 613, 669	2.11 (2.03)
**PF** ^**+**^ **-(OMe)**_**3**_	10 mM TFA	4.1	378, 467, 478	361, 448, 464	504	– ^f^ (2.38) ^f^
	No TFA	2.4	345, 478, 456, 531, 618	337, 419, 534, 572	505, 618, 672	2.08 (2.02)
**PF** ^**+**^ **-Me**	10 mM TFA	99.2	358, 451, 482	360, 446, 464	500	2.57 (2.46)
	No TFA	7.4	336, 422, 529	328, 417, 531	499, 612, 669	2.11 (2.02)
**PF** ^**+**^ **-F**	10 mM TFA	66	354, 398, 448	355, 388, 448	505	2.57 (2.48)
	No TFA	6.6	324, 422, 531	325, 417, 531	505, 614, 672	2.11 (2.02)
**PF** ^**+**^ **-H**	10 mM TFA	74.1	353, 402, 445	353, 409, 442	506	2.57 (2.47)
	No TFA	3.6	325, 428, 520	324, 421, 519	614, 668	2.11 (2.00)
**PF** ^**+**^ **-Py**	10 mM TFA	31.7	349, 404, 447	350, 410, 448	515	2.55 (2.49)
	No TFA	2.9	323, 421, 535	322, 418, 534	619, 677	2.17 (2.01)
**PF** ^**+**^ **-CN**	10 mM TFA	20.7	353, 412, 450	354, 422, 452	522	2.51 (2.42)
	No TFA	2.9	328, 429, 524	328, 424, 528	626, 663	2.07 (2.01)
**PF** ^**+**^ **-NO**_**2**_	10 mM TFA	32.2	353, 456, 476	360, 458, 472	515	– ^f^ (2.11)
	No TFA	2	314, 389, 531	320, 427, 534	620, 667	– ^f^ (1.91)

a Fluorescence quantum yields determined using fluorescein in aqueous media (pH = 10) as a standard, *λ*_*ex*_ = 465 nm, unless otherwise noted.

b Absorption maxima.

c Maxima of the excitation spectra, in the presence of 10 mM TFA: *λ*_*em*_ = 515 nm; and in the absence of TFA: *λ*_*em*_ = 615 nm.

d Maxima of the emission spectra, *λ*_*ex*_ = 465 nm.

e Zero-to-zero energy, $E_{00}$, obtained from the crossing point of normalized optical absorption and fluorescence spectra that are TDM-corrected [60]. Values in parentheses are obtained from the bathochromic edge of the optical absorption spectra.

f The optical absorption and excitation spectra do not match in their bathochromic regions, hence, the crossing point does not represent the energy between the ground and the lowest singlet excited state.

**Table 2.** Reduction potentials of the **PF**^**+**^**-X** derivatives (Scheme 1), along with their corresponding energies, E, of photooxidation and photoreduction propensities.

	$E_{{\text{PF}}^{+}|{\text{PF}}^{•}}^{\left(1/2\right)}/\text{V}$ vs. SCE ^a^	$E_{{}^{1}{\left({\text{PF}}^{+}\right)}^{*}|{\text{PF}}^{•}}^{\left(1/2\right)}/\text{V}$ vs. SCE (E/eV vs. Vacuum) ^b,c^	$E_{{\text{PF}}^{•+}|\text{PF}}^{\left(1/2\right)}/\text{V}$ vs. SCE ^d^	$E_{{\text{PF}}^{•+}{|}^{1}{\left(\text{PF}\right)}^{*}}^{\left(1/2\right)}/\text{V}$ vs. SCE (E/eV vs. Vacuum) ^e,f,g^
**PF** ^**+**^ **-NMe**_**2**_	−0.607 ± 0.003 ^h^	1.34 (−6.02)	0.967 ± 0.052	−0.973 (−3.71)
**PF** ^**+**^ **-OMe**	−0.497 ± 0.007 ^g^	1.95 (−6.63)	1.05 ± 0.06	−1.06 (−3.62)
**PF** ^**+**^ **-(OMe)**_**3**_	−0.480 ± 0.006	1.90 (−6.58)	1.06 ± 0.03	−1.02 (−3.66)
**PF** ^**+**^ **-Me**	−0.444 ± 0.006	2.13 (−6.81)	0.966 ± 0.056	−1.14 (−3.54)
**PF** ^**+**^ **-F**	−0.422 ± 0.032 ^g^	2.15 (−6.83)	0.983 ± 0.020	−1.23 (−3.55)
**PF** ^**+**^ **-H**	−0.398 ± 0.063	2.17 (−6.85)	1.12 ± 0.04	−0.990 (−3.69)
**PF** ^**+**^ **-Py**	−0.349 ± 0.008	2.20 (−6.88)	1.19 ± 0.05	−0.980 (−3.70)
**PF** ^**+**^ **-CN**	−0.339 ± 0.006	2.17 (−6.85)	1.27 ± 0.09	−0.800 (−3.88)
**PF** ^**+**^ **-NO**_**2**_	−0.232 ± 0.005 ^g^	1.89 (−6.56)	1.15 ± 0.03	−0.760 (−3.92)

a For acetonitrile, in the presence of 50 mM (*n*-C_4_H_9_)_4_NPF_6_ and 1 mM TFA, except for **PF**^**+**^**-NMe**_**2**_ where no TFA was added (Figure 5).

b The values of $E_{{}^{1}\left({\text{PF}}^{+}\right)*|{\text{PF}}^{•}}^{\left(1/2\right)}=E_{{\text{PF}}^{+}|{\text{PF}}^{•}}^{\left(1/2\right)}+F^{-1}\;E_{00}$ (*F* is the Faraday constant) correspond to rough estimates of the reduction potentials of the singlet excited states of the **PF**^**+**^**-X** dyes, considering the following: (1) an assumption that the measured half-wave potentials, *E*^(1/2)^, and the estimated $E_{00}$ for samples with 1 to 10 mM TFA are representative of the acidic forms of the dyes; (2) the values of *E*^(1/2)^ are for MeCN in the presence of 50 mM supporting electrolyte and the values for $E_{00}$ are for neat MeCN; and (3) $E_{{}^{1}\left({\text{PF}}^{+}\right)*|{\text{PF}}^{•}}^{\left(1/2\right)}$ does not account for the Born-solvation term (Δ*G*_*S*_) or any contribution of the Coulombic-work term (*W*) in the Rehm–Weller equation [38,82].

c The values in the parentheses represent the propensity of the protonated dyes to photooxidize, expressed vs. the vacuum level of an electron at rest that, according to the Koopmans theorem, relates to the energy levels of the HOMOs, i.e., $E=-4.68-F\;E_{{}^{1}\left({\text{PF}}^{+}\right)*|{\text{PF}}^{•}}^{\left(1/2\right)}$ [83].

d For acetonitrile, in the presence of 50 mM (*n*-C_4_H_9_)_4_NPF_6_ and absence of TFA.

e The values of $E_{{\text{PF}}^{•+}{|}^{1}\left(\text{PF}\right)*}^{\left(1/2\right)}=E_{{\text{PF}}^{•+}|\text{PF}}^{\left(1/2\right)}-F^{-1}\;E_{00}$ correspond to rough estimates of the reduction potentials of the oxidized deprotonated dyes, **PF-X**, for the excited state.

f The values in the parentheses represent the propensity of the deprotonated dyes to photoreduce that, according to the Koopmans theorem, relates to the energy levels of the LUMOs, i.e., $E=-4.68-F\;E_{{\text{PF}}^{•+}{|}^{\text{1}}\left(\text{PF}\right)*}^{\left(1/2\right)}$

g Due to the irreversible reduction revealed by the cyclic voltammograms, i.e., the absence of anodic peaks (Figure 3), the half-wave potentials were obtained from the inflection points of the cathodic waves [84,85].

h The reported value is for samples with no THF added. Adding THF to the **PF**^**+**^**-NMe**_**2**_ samples shifts the measured reduction potentials to −0.5 V vs. SCE, which is consistent with protonation of the dimethylamine making ring E in the dication, **PF**^**+**^**-NH**^**+**^**Me**_**2**_, more electron deficient than for **PF**^**+**^**-NMe**_**2**_. The fluorescence spectra confirm the presence of small amounts of the dication (Figure 2e) and the fast proton transfer can induce shifts in the recorded cathodic waves that is enhanced in acidic media.
